# Asthma Intervention with Residential Ventilation and Air Cleaner (AIRVAC) Study: A 4-arm parallel-group randomized controlled trial protocol

**DOI:** 10.1371/journal.pone.0358221

**Published:** 2026-09-25

**Authors:** Insung Kang, Semi Park, Yidan Zhang, Steven Tanner McCullough, Tia LaFavor, Yike Shen, June Young Park

**Affiliations:** 1 Department of Civil Engineering, University of Texas at Arlington, Arlington, Texas, United States of America; 2 Department of Earth and Environmental Sciences, University of Texas at Arlington, Arlington, Texas, United States of America; 3 Rebuilding Together North Texas, Plano, Texas, United States of America; Southeast University, CHINA

## Abstract

**Background:**

Evidence linking exposure to airborne particulate and gaseous pollutants, as well as mold spores, with asthma control and severity is well established. A growing body of literature has demonstrated that ventilation and air filtration can improve indoor air quality (IAQ); however, clinical evidence on their comparative health impacts remains limited. Therefore, this trial aims to evaluate the long-term effectiveness of energy recovery ventilators (ERVs) and portable air cleaners (PACs) in reducing indoor air pollutants and mold levels and improving health outcomes in individuals with asthma. Secondary objectives includes associations between housing conditions, occupant behaviors, indoor environmental exposures, and asthma-related health outcomes.

**Methods:**

The AIRVAC study is a single-blind, placebo-controlled, 4-arm, parallel-group, randomized controlled trial, with a pre-intervention period of up to 1 year and a 1-year post-intervention period. Children aged 5–17 years and adults with physician-diagnosed asthma will be recruited from more than 80 households across the Dallas-Fort Worth (DFW) area in Texas. Following the pre-intervention period, each household will be randomized to receive one of four different interventions midway through the study: (1) active ERVs, (2) sham ERVs, (3) active PACs, and (4) sham PACs. Primary health outcomes include asthma control; secondary outcomes include pulmonary function, asthma-related quality of life, stress, and sleep quality. Initial housing assessments will be conducted prior to the pre-intervention period to characterize occupant behaviors and housing-related factors that may contribute to asthma exacerbations and affect ERV installation. Environmental exposures will be assessed by combining measurements of indoor and outdoor air pollutants, including particulate matter (PM), nitrogen dioxide (NO_2_), carbon monoxide (CO), and volatile organic compounds (VOCs), as well as temperature, relative humidity, and indoor and outdoor mold, using low-cost and research-grade air quality sensors and mold sampling methods.

**Discussion:**

Findings from the AIRVAC study will provide important evidence on the comparative effects of residential ventilation and air cleaning interventions on IAQ, mold, and asthma-related health outcomes among children and adults with asthma. The study will also examine the relationships among housing characteristics, occupant behaviors, indoor environmental exposures, and asthma-related health outcomes, while providing insights into the operation and performance of residential ventilation and air cleaning interventions in vulnerable households.

## Introduction

Asthma is an allergic respiratory disease characterized by airway inflammation, mucus accumulation, and bronchoconstriction, resulting in reduced airflow. Common symptoms include wheezing, shortness of breath, chest tightness, and coughing, which can range in severity from mild to severe [1,2]. Asthma affects approximately 300 million people worldwide [3], and nearly 27 million Americans have been diagnosed with asthma, including 4.5 million children and 22.3 million adults [4]. The annual economic burden of asthma in the United States is estimated to be approximately $82 billion, including medical care, medications, hospitalizations, lost work and school days, and mortality-related costs [5].

Air pollution is one of the major triggers and determinants of asthma, influencing both disease onset and exacerbation across age groups, particularly in residences where people spend most of their time indoors [6]. Exposure to pollutants such as particulate matter (PM), nitrogen dioxide (NO_2_), volatile organic compounds (VOCs), and carbon monoxide (CO), induces oxidative stress and airway inflammation that lead to epithelial injury, bronchial hyperresponsiveness, and airway remodeling [7–10]. Particularly in residences where individuals spend most of their time indoors, combustion sources such as gas stoves, tobacco smoke, and unvented heating appliances can generate pollutant concentrations that exceed acute or chronic health standards, resulting in asthma attacks and exacerbations [11–14]. Further, indoor dampness and mold can exacerbate asthma through the release of spores and mycotoxins from species such as *Aspergillus* and *Cladosporium*, which disrupt epithelial integrity and provoke IgE-mediated and Th2-driven inflammation [15,16].

Ventilation and air cleaning are widely recognized and well-established strategies for effectively reducing indoor air pollutants when controlling or eliminating the source of the pollutants is impractical as a remedial measure [17]. Balanced ventilation systems, often integrated with energy recovery ventilators (ERVs), simultaneously introduce filtered outdoor air and exhaust stale indoor air, diluting both particulate and gaseous pollutants while maintaining neutral air pressure within the building for optimal air exchange. ERVs can be equipped with Minimum Efficiency Reporting Value (MERV) 8 or higher filters to remove particles, allergens, and other contaminants from incoming outdoor air. Air cleaners, also known as stand-alone air filtration systems, primarily remove airborne pollutants from indoor air. High-efficiency particulate air (HEPA) filtration can remove at least 99.97% of particles with diameters of 0.3 µm, while gas-phase filtration (e.g., activated carbon filters) can adsorb some gaseous pollutants, including VOCs and odors.

Substantial empirical evidence supports the effectiveness of mechanical ventilation systems in improving indoor air quality (IAQ) in homes [18–25]. Although several clinical studies have further investigated the effects of residential ventilation systems on asthma-related health outcomes [26–31], none of these studies comprehensively assessed IAQ, including particulate and gaseous pollutants, mold, as well as temperature and humidity, and only one trial implemented a true placebo control; the others either provided no intervention to controls or had no control group. Similarly, a growing body of literature has shown that residential HEPA air cleaners can lower indoor PM and allergens and yield at least modest health benefits for a variety of populations, including individuals with asthma [17,32,33]. However, important knowledge gaps remain, including small sample sizes, short-term intervention periods, and variability in outcome measures.

### Objectives and hypotheses

The primary objective of this study is to evaluate the comparative long-term (one-year) effects of ERVs and portable HEPA air cleaners on reducing exposure to indoor particulate and gaseous pollutants and mold, as well as on improving asthma-related health outcomes in inner-city children and adults with asthma in the Dallas-Fort Worth (DFW) area of Texas. We hypothesize that year-long use of ERVs and portable HEPA air cleaners will lead to improvements in IAQ and asthma-related health outcomes during the post-intervention period compared to the pre-intervention period, whereas control groups are expected to show no significant changes. Further, we hypothesize that two interventions will differ in both the profiles and magnitudes of IAQ and health improvements, as ERVs are designed to reduce both particulate and gaseous pollutants and help control indoor humidity to lower the risk of mold growth, whereas HEPA air cleaners primarily remove airborne particles from indoor air.

## Methods

The analysis and reporting of trial findings will adhere to the Consolidated Standards of Reporting Trials (CONSORT) extension for cluster randomized trials and will be guided by a pre-specified statistical analysis plan. The Asthma Intervention with Residential Ventilation and Air Cleaner (AIRVAC) study was approved by the Institutional Review Board (IRB) of the University of Texas at Arlington (UTA) Office of Regulatory Services (IRB #2025-0280; approval date: 08/01/2025). Written informed consent will be obtained from all participants (or their legal guardians, as applicable) prior to enrollment and any study procedures during the initial home visit (V0), in accordance with institutional ethical guidelines. Participant confidentiality will be maintained by assigning unique study identification numbers to participants and securely storing all study data with access limited to authorized research personnel. The study is registered at ClinicalTrials.gov (NCT07196436; registration date: 9/29/2025). The recruitment period for this study is from September 2025 to September 2026; recruitment is ongoing at the time of writing. Data collection has not yet begun and is expected to be completed by February 2028, with study results anticipated in April 2028.

### Study design

The AIRVAC study is a single-blind, placebo-controlled, four-arm, parallel-group, randomized controlled trial ERVs and portable HEPA air cleaners, with a pre-intervention period of up to 1 year and a 1-year post-intervention period. The pre-intervention period was designed to characterize baseline variability in indoor environmental conditions and health outcomes across seasons and to account for seasonal variations in longitudinal analyses using time-related covariates. Participants will be randomly assigned to one of four groups: (1) active ERV, (2) sham (placebo) ERV, (3) active portable HEPA air cleaner, or (4) sham (placebo) portable HEPA air cleaner.

The study involves five home visits. During the initial home visit (V0), the field team, which includes a BPI Building Analyst Professional (BAP)-certified investigator [34] and a licensed HVAC contractor, will conduct initial home visits (V0) to obtain information on housing conditions, health and safety concerns, and potential asthma triggers that may contribute to exacerbations or affect ERV installation. Key parameters to be assessed include home size, construction type, heating, ventilation, and air conditioning (HVAC) system, window operation, kitchen stove and hood, occupant behaviors, and other environmental risk indicators (e.g., visible mold, water damage, asbestos, chipping paint, pests, and pets). Additionally, for homes deemed eligible following the health and safety evaluation, blower door tests will be performed to measure the airtightness of the building envelope.

**Fig 1:**
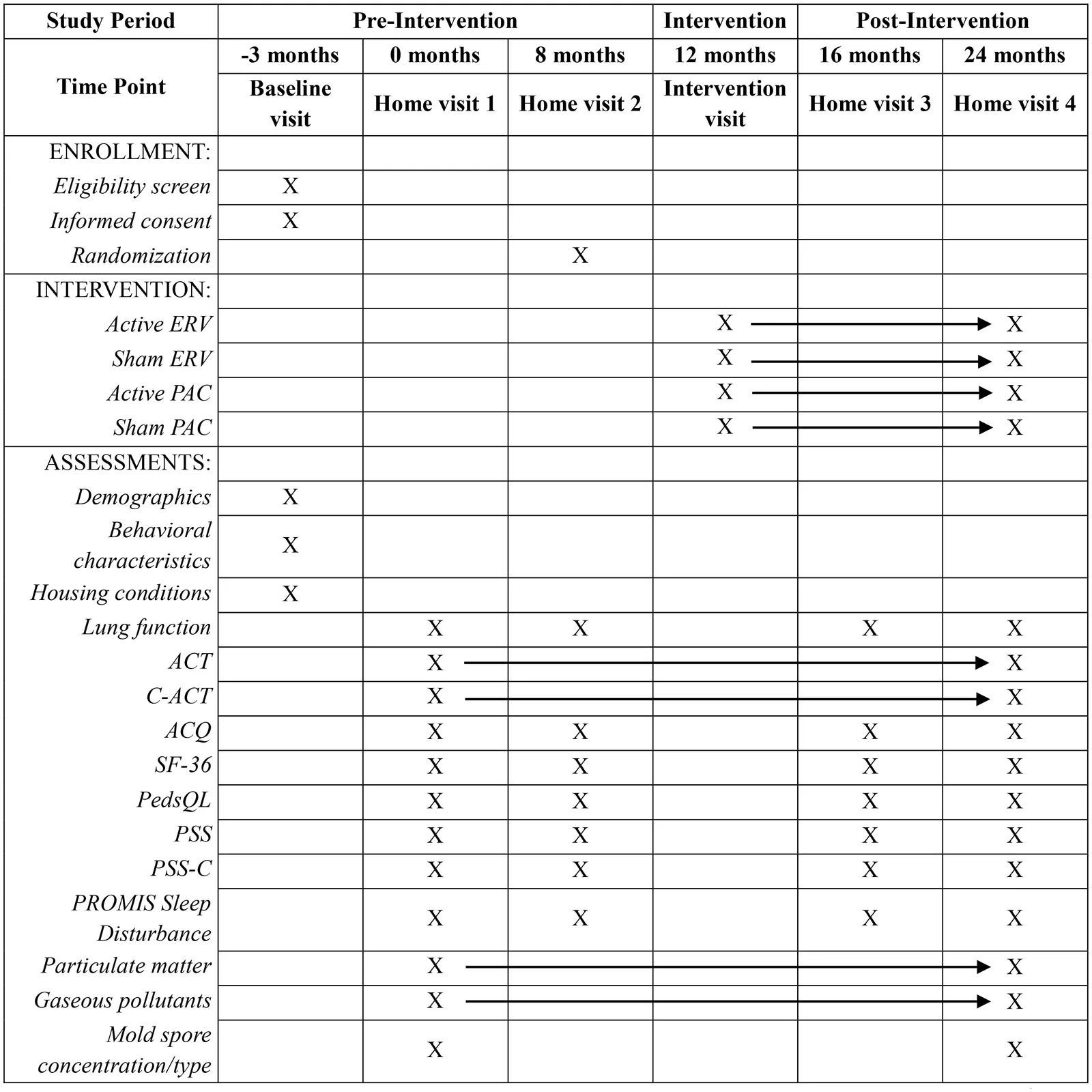
SPIRIT schedule of enrollment, interventions, and assessments. ERV, energy recovery ventilator; PAC, portable air cleaner; ACT, asthma control test; C-ACT, childhood asthma control test; ACQ, asthma control questionnaire; SF-36, 36-item short form health survey; PedsQL, pediatric quality of life inventory; PSS, perceived stress scale; PSS-C, perceived stress scale–child; PROMIS, patient-reported outcomes measurement information system.

Following the initial visit, four follow-up visits (V1-V4) will be conducted to assess environmental exposures and health outcomes (two during the pre-intervention period and two during the post-intervention period). Fig 1 shows an overview of the study visit timeline with tasks that will be performed at each visit.

### Participant recruitment

The target study population includes children aged 5–17 years and adults aged 18 years and older with asthma from 80 households. Initially, approximately 88 households will be recruited to account for potential dropout and ensure that the required sample size is maintained throughout the study period. Participants will be primarily recruited from applicants to Rebuilding Together North Texas (RTNTX), a nonprofit organization that administers the Safe & Healthy Homes program. This program provides essential home repairs, safety improvements, and accessibility modifications for low-income residents across diverse communities in the DFW area.

Eligibility requirements for participation in the study include: (1) homes must have at least one occupant with self-reported physician-diagnosed asthma; (2) homes must be non-smoking; (3) homes must be owner-occupied; and (4) participants must agree to participate in the entire 2-year study duration and allow for five home visits by the research team. Potentially eligible participants will complete an electronic screening form prior to enrollment, based on the inclusion and exclusion criteria listed below.

The inclusion criteria:

- Age: children 5–17 years; adults ≥18 years.
- Individuals with physician-diagnosed asthma.
- Residency in the DFW area.
- English speaking and writing.
- Able to provide consent.
- Able to communicate regularly by phone.

The exclusion criteria:

- Homes with any current smokers
- Anticipated relocation outside the DFW area within the two-year study period.
- Hazardous conditions and/or safety concerns in or around the participant’s home.
- Use of any device not provided as part of the study, including ERVs or portable HEPA air cleaners, in the participant’s home at any time during the trial.

Participant engagement will be maintained through home visits and monthly asthma surveys via phone. Primary participants will receive a total of $200 in gift cards across the four follow-up visits (V1-V4), and additional eligible family members may receive $100 each. Participants will have the option to keep the intervention device; those assigned to a sham device will receive an active device at study completion. Participants who do not complete required assessments will not be randomized or remain in the study. Compensation is contingent upon completing all study procedures, and participants who withdraw or fail to complete visits will forfeit subsequent payments.

### Interventions

The AIRVAC study will involve ERV and HEPA air cleaner interventions aimed at improving IAQ and asthma-related health outcomes. Following the pre-intervention period, participants will be randomized to receive one of four interventions during the 1-year post-intervention period: (1) active ERV, (2) sham (placebo) ERV, (3) active portable HEPA air cleaner, or (4) sham (placebo) portable HEPA air cleaner.

Due to the specific housing conditions required for ERV installation, including accessible ductwork for supply and exhaust, adequate space and access for the ERV unit, electrical safety, and proper sealing of penetrations, a constrained randomization approach will be employed. Prior to randomization, each home will undergo an ERV installation feasibility assessment based on the information collected during the initial walk-through visit (V0). Homes that meet the ERV installation criteria will be eligible for assignment to ERV or portable air cleaner study arms, whereas homes that do not meet the ERV installation criteria will only be eligible for portable air cleaner study arms.

After intervention eligibility is determined, participants will be randomly assigned to the available active or sham groups using a computer-generated randomization sequence in Stata (v.19) [35]. Stratified randomization will be used to maintain balance across the four study arms within predefined strata based on key housing and participant characteristics. Housing characteristics will include building envelope air leakage measured by blower door testing, home size, and exhaust fans, while participant characteristics will include baseline health status. The randomization sequence will be generated and securely maintained by the PIs. Allocation assignments will remain concealed until completion of eligibility confirmation and baseline assessments. All participants will remain blinded to whether they receive the active or sham intervention throughout the entire study duration. To maintain participant blinding, all field research staff and HVAC contractors will be trained not to disclose intervention assignments to participants.

For the 40 homes assigned to the ERV intervention, we will install a Broan-NuTone B160E75RS/T units. The required airflow rate of each unit will be determined based on the floor area and number of bedrooms, in accordance with ASHRAE Standard 62.2–2025, *Ventilation and Acceptable Indoor Air Quality in Residential Buildings* [36]. The building envelope leakage measured during the initial home assessments will be applied as infiltration credits in the ventilation rate calculations to account for natural air leakage. The ERV units will then operate continuously at the specified ventilation rate throughout the intervention period.

Sham ERVs will be installed in the same manner as active units but will be programmed to operate in recirculation mode, without supplying outdoor air or altering ventilation rates. They will draw indoor air from the home and return it without any dilution or exchange with outdoor air, as the outdoor air intake damper remains closed. Participants will not have access to the control panel settings during the study period. This modification will maintain participant blinding, as occupants will experience airflows and fan noise similar to those of active ERVs.

Forty (40) homes in the air cleaner group will receive a Medify Air MA-112 unit, primarily placed in the living room (or in the bedroom if occupants spend the majority of their time). In cases where additional eligible family members reside in the same household, an additional Medify Air MA-50 unit will be deployed in their bedrooms. Both air cleaners are equipped with HEPA and activated carbon filters. In homes assigned to the sham control group, filters will be removed from the air cleaner units. Both active and sham air cleaner units will be sealed and have identical external appearance and installation configurations to maintain participant blinding.

During scheduled home visits in the post-intervention period, field staff will check the ERV and air cleaner units to verify proper operation and confirm that blinding measures are maintained, including ensuring that ERV control panel settings remain unchanged and inaccessible to participants and that air cleaner unit seals remain intact.

### Environmental exposure assessments

The study will involve the measurements of indoor criteria air pollutants, mold, and environmental conditions, including temperature and relative humidity (T/RH) to assess the magnitude of exposures in homes. Research-grade air quality sensors will be deployed for approximately one week during the four scheduled visits (V1–V4). The TSI Q-Trak XP IAQ monitor (7585) and DustTrak aerosol monitor (8530) will be placed alongside the indoor low-cost sensors to provide accurate measurements of size-resolved PM, NO_2_, NO, CO, O_3_, and formaldehyde (HCHO).

Additionally, low-cost sensors will continuously collect indoor and outdoor air quality data over the two-year study period. A total of 160 sensors will be fabricated using a microcontroller (Raspberry Pi 4 Model B, 4 GB) and multiple pollutant sensors, including a Sensirion SPS30 for PM, an Adafruit SGP30 for VOCs, an Amphenol MiCS-2714 for NO_2_, and a Sensirion SCD4x for CO_2_ and T/RH, with an estimated cost of approximately $150 per unit. Those economically viable sensor selections are verified in a similar study to measure indoor environmental quality for residential buildings [37]. The low-cost air quality sensors will be deployed indoors (primarily in the living room) and outdoors (primarily in the backyard or porch area) during the first follow-up visit (V1) and will remain in place throughout the study. For each visit, the research team will log the senor readings to check the quality of data collection with proper tech maintenance on the sensors. Long-term time-series data from these sensors will be calibrated against short-term (approximately 1-week) high-accuracy measurements measured by research-grade instruments, and additional details on the calibration process are provided in the Statistical Analysis Plan section.

Moreover, the study will involve mold assessments through spore-trap analysis of air samples, direct microscopic examination of surface samples, and fungal DNA sequencing of dust samples. Indoor and outdoor air samples will be collected using an Air-O-Cell sampling cassette with a calibrated Bio-Pump (Environmental Express), surface samples with sterile cotton-tipped swabs, and dust samples from HVAC filters and/or damp areas with visible mold or water damage (e.g., kitchen and bathroom) in each home at V1 and V4. Air and surface samples will be submitted to the accredited laboratory Eurofins USA for spore-trap and direct microscopic analyses. The dust samples will be sent to Co-PI Shen’s lab for storage, DNA extraction, followed by fungal Internal Transcribed Spacer (ITS) sequencing at the North Texas Genome Center at UTA within three months of sample collection completion.

### Health outcome assessments

#### Primary outcome measures.

We will use the following measures to assess asthma control:

- Asthma Control Test (ACT): Monthly ACT surveys will be administered electronically using an online survey link distributed to participants. The ACT is a 5-item, clinically validated questionnaire designed to assess the multi-dimensional nature of asthma control, including asthma symptoms, use of rescue medications, and the impact of asthma on daily functioning [38]. ACT scores range from 5 (“poor control of asthma”) to 25 (“complete control of asthma”), with a cut-off of 19 used to identify participants with suboptimal asthma control according to the Global Initiative for Asthma (GINA) guidelines: a score >19 indicates “well-controlled asthma,” whereas a score ≤19 indicates “poorly controlled asthma” [39,40]. Children under 12 years of age will complete the Childhood Asthma Control Test (C-ACT).
- Asthma Control Questionnaire (ACQ): In addition to monthly ACT, we will administer the ACQ during the home visits (V1-V4) to assess asthma control more comprehensively. The ACQ consists of 7 items covering asthma symptoms, rescue bronchodilator use, and lung function (FEV_1_, forced expiratory volume in one second, FEV_1_). Each scored on a 7-point scale from 0 (“no impairment”) to 6 (“maximum impairment”), and the overall score is calculated as the mean of all items, with lower scores indicating better control. Consistent with established guidelines, an ACQ score ≤0.75 will be used to identify “well-controlled asthma,” whereas a score ≥1.50 will indicate “poorly controlled asthma,” with intermediate values reflecting partially controlled asthma [41,42].

#### Secondary outcome measures.

Additionally, pulmonary function, health-related quality of life, perceived stress, and sleep quality will be assessed during each home visit (V1-V4):

- Pulmonary Function Testing (PFT): PFT will be conducted by a trained research staff during the home visits (V1-V4). A portable spirometer will be used to measure forced expiratory volume in 1 second (FEV_1_), forced vital capacity (FVC), and the FEV_1_/FVC ratio, following standardized procedures. Participants will perform at least three acceptable maneuvers per visit, and the highest values will be recorded for analysis.
- Health-related quality of life will be assessed using the Short Form Health Survey (SF-36) for adults and the Pediatric Quality of Life Inventory (PedsQL) for children and adolescents. The SF-36 is a 36-item, validated instrument assessing eight health domains: physical functioning, role physical, bodily pain, general health, vitality, social functioning, role emotional, and mental health. Physical and Mental Component Summary scores (PCS and MCS) will be calculated as standardized T-scores (mean 50, SD 10), where higher scores indicate better overall physical or mental health [43]. The PedsQL will be administered using age-appropriate generic core versions for young children (ages 5–7), children (8–12), and adolescents (13–18). Each version consists of 23 items assessing physical, emotional, social, and school functioning. Responses are transformed to a 0–100 scale, with higher scores indicating better health-related quality of life [44].
- Perceived stress will be assessed using the 10-item Perceived Stress Scale (PSS) for adults and the 14-item child version (PSS‑C) for children and adolescents aged 5–18 years. Both instruments measure the degree to which individuals perceive their lives as unpredictable, uncontrollable, and overloaded. Items are rated on a 5-point Likert scale (0 = “never” to 4 = “very often”), with total scores ranging from 0–40 for the PSS and 0–56 for the PSS-C; higher scores indicate greater perceived stress [45,46].
- Sleep quality will be assessed using age-appropriate versions of the PROMIS Sleep Disturbance short form, which evaluates self-reported sleep quality, difficulties with initiating or maintaining sleep, restorative sleep, and overall sleep-related satisfaction over the past week. Raw scores range from 8 to 40, with higher T-scores indicating greater sleep disturbance (i.e., poorer sleep quality) [47,48].

### Power calculations

The sample size and statistical power for this 4-arm parallel-group randomized controlled trial is analyzed for various scenarios based on a range of likely magnitudes of outcomes for asthma-related health outcome and environmental exposure assessments (Table 1). Calculations are made utilizing a robust statistical software tool, G*Power ver. 3.1.9.7, for a repeated measures ANOVA with a within-between interaction [49]. A range of effect sizes from small (Cohen’s f = 0.10), medium (f = 0.25), to large (f = 0.40) are considered to accommodate varying expected intervention effects, ensuring both robustness and generalizability of the findings [50]. Sample size calculations were conducted for the health outcomes (ACT and PFT) and environmental outcomes (IAQ and mold measurements). Required sample sizes were estimated based on expected effect sizes to achieve a statistical power >0.80 and adjusted for a 10% dropout rate. For monthly ACT surveys (n = 24) and monthly average low-cost IAQ measurements (n = 24), the required sample sizes ranged from 84 households for a small effect size (0.10) to 9 households for a large effect size (0.40). For PFT and mold measurements, the required sample sizes were estimated at 220 and 308 households, respectively, for detecting a small effect size (0.10); 40 and 53 households, respectively, for a medium effect size (0.25); and 18 and 26 households, respectively, for a large effect size (0.40). Although larger sample sizes were required to detect a small effect size (0.1) for PFT and mold, these estimates represent the sample sizes needed to detect very small changes. Further, the AIRVAC study was primarily designed to evaluate intervention effects on asthma outcomes and IAQ, for which the estimated sample sizes were within the planned recruitment range. Therefore, the final target sample size was set at 80 households, with an initial recruitment target of 88 households to account for potential dropout.

**Table 1:** Sample size calculations.

Effect Size (Cohen’s f for ANOVA)	Small			Medium			Large
	0.1	0.15	0.2	0.25	0.3	0.35	0.4
**Asthma Control Test (ACT) Surveys & IAQ Measurements (No. of measurements = 24)**							
Statistical Power	0.82	0.83	0.90	0.89	0.90	0.98	0.92
Sample Size Per Group	19	9	6	4	3	3	2
Total Sample Size	76	36	24	16	12	12	8
Total Sample Size (adjusted for 10% dropout)	**84**	40	26	**18**	13	13	**9**
**Pulmonary Function Testing (No. of measurements = 4)**							
Statistical Power	0.8	0.81	0.8	0.83	0.86	0.83	0.82
Sample Size Per Group	50	23	13	9	7	5	4
Total Sample Size	200	92	52	36	28	20	16
Total Sample Size (adjusted for 10% dropout)	**220**	101	57	**40**	31	22	**18**
**Mold Measurements (No. of measurements = 2, baseline & end-line)**							
Statistical Power	0.81	0.81	0.82	0.8	0.82	0.83	0.86
Sample Size Per Group	70	32	19	12	9	7	6
Total Sample Size	280	128	76	48	36	28	24
Total Sample Size (adjusted for 10% dropout)	**308**	141	84	**53**	40	31	**26**

Sample sizes were calculated based on repeated measures ANOVA (α = 0.05, power = 0.80) and expected effect sizes, with adjustment for a 10% dropout rate.

### Data management and statistical analysis plan

The full data collection will include three categories of data: (1) human subject data, including demographic and socioeconomic characteristics and asthma-related health outcomes collected via baseline and follow-up questionnaires; (2) housing characteristics, including building and dwelling information; and (3) environmental exposure data, including indoor and outdoor air quality and mold measurements. All data will be de-identified prior to analysis to protect participant confidentiality, with direct identifiers removed before sharing. Data will be stored as comma-separated text files and spreadsheet files on a secure central server in the PI’s laboratory and processed electronically using Stata or Python in appropriate analysis formats. De-identified data will be shared only with the PI. Missing data will be handled using multiple imputation under the missing-at-random assumption, where collected data will be used to predict missing values. At least 20 imputed datasets will be generated, and convergence diagnostics and comparisons between observed and imputed data will be performed to assess plausibility [51]. Mixed-effects models for repeated measures will be used to account for within-subject correlations, and sensitivity analyses will compare complete-case and multiple imputation results.

Descriptive statistics will summarize participant demographics (e.g., age, sex, race/ethnicity, household income) and study variables by group, with continuous variables presented as means, medians, standard deviations, interquartile ranges (IQRs), or other distributional parameters, and categorical variables as frequencies and percentages. Appropriate statistical tests will be applied based on data type and distribution, including paired and unpaired t-tests, Wilcoxon signed-rank or rank-sum tests, and chi-square tests for categorical comparisons.

The primary analyses will follow the intention-to-treat (ITT) principle, including all randomized households analyzed according to their assigned intervention group, to evaluate the effects of ERVs and portable HEPA air cleaners on IAQ, mold reduction, and asthma-related health outcomes by comparing the intervention and control (sham) groups using repeated measures analysis of variance (ANOVA) with a within-between interaction. Pairwise comparisons will be conducted between each intervention and control group (Group 1 vs. Group 2, Group 3 vs. Group 4) and between intervention groups (Group 1 vs. Group 3), using Tukey’s Honest Significant Difference (HSD) test to adjust for multiple comparisons. Repeated monthly ACT scores will be analyzed using multivariate mixed-effects models to account for within-subject correlations over time. Per-protocol (PP) analyses will be conducted as sensitivity analyses and will include only households that adequately adhered to the assigned intervention protocol. Intervention adherence will be assessed using plug load logger data for both ERV and air cleaner units, including device operation status and operating duration [52]. Additionally, subgroup analyses will be conducted to explore whether intervention effects differ by demographic and socioeconomic characteristics, baseline asthma severity, and baseline IAQ.

For secondary analyses, multivariate linear and logistic regression models will examine associations among housing characteristics, occupant behaviors, indoor environmental exposures (e.g., air pollutants and mold), and asthma-related health outcomes. Housing characteristics will be considered as potential covariates or confounders, when applicable, based on biological plausibility and statistical considerations. Prior to multivariable modeling, correlations among environmental exposure variables (e.g., PM, NO_2_, VOCs, and mold indices) will be evaluated using Pearson or Spearman correlation coefficients, depending on data distribution. Variance inflation factors (VIFs) will also be calculated to assess multicollinearity among predictors. Highly correlated exposures will not be included simultaneously in the same model to minimize multicollinearity, overfitting, and unstable estimates.

Lastly, indoor and outdoor air quality data collected by low-cost sensors deployed throughout the study will be validated and calibrated against co-located research-grade monitors. Paired measurements from low-cost sensors and reference instruments will be used to develop calibration models. Linear regression will be used to estimate calibration coefficients, including slope and intercept, which will be applied to adjust low-cost sensor measurements for sensor bias and drift over time. Sensor performance will be assessed through quality assurance checks, including evaluation of data completeness, abnormal readings, and consistency with reference measurements. The calibrated data will subsequently be used in all analyses described above to ensure accuracy and consistency. All analyses will be conducted in Stata (v.19) [35] and/or R (v.4.5.2) [53] by multiple team members to minimize bias. Detailed procedures for sensor calibration and validation, including co-location with research-grade instruments and development of calibration models, are described in our previous calibration report [18].

The trial does not involve a Data Monitoring Committee (DMC) as the interventions (i.e., ERV and PAC) are non-pharmacological environmental interventions with minimal expected risk. Study oversight will be conducted by the PIs, who will monitor study progress, data quality, and participant safety throughout the trial. Any unexpected problems or adverse events, if they occur, will be documented and reported to the IRB in accordance with institutional requirements. The statistical analysis plan will be finalized prior to the final data lock and database unblinding. Any deviations from the prespecified analysis plan will be documented and reported transparently.

### Knowledge dissemination

Key findings from the final analyses will be disseminated through peer-reviewed publications in environmental and public health journals and presented at academic conferences and seminars. Results will also be shared with local communities, healthcare providers, policymakers, and the public using appropriate communication channels. Paper records will be stored in a locked file cabinet in the PI’s office, accessible only to authorized personnel. All electronic data will be securely stored and shared via UTA’s encrypted, password-protected OneDrive system.

## Supporting information

S1 Checklist SPIRIT 2025 editable checklist. (DOCX)
